# Optimization for Pipeline Corrosion Sensor Placement in Oil-Water Two-Phase Flow Using CFD Simulations and Genetic Algorithm

**DOI:** 10.3390/s23177379

**Published:** 2023-08-24

**Authors:** Shuomang Shi, Baiyu Jiang, Simone Ludwig, Luyang Xu, Hao Wang, Ying Huang, Fei Yan

**Affiliations:** 1 Department of Civil, Construction, and Environmental Engineering, North Dakota State University, Fargo, ND 58105, USA; shuomang.shi@ndsu.edu (S.S.); luyang.xu@ndsu.edu (L.X.); fei.yan@ndsu.edu (F.Y.); 2 Department of Civil and Environmental Engineering, School of Engineering, Rutgers, The State University of New Jersey, New Brunswick, NJ 08901, USA; bj224@scarletmail.rutgers.edu (B.J.); hw261@soe.rutgers.edu (H.W.); 3 Department of Computer Science, North Dakota State University, Fargo, ND 58105, USA; simone.ludwig@ndsu.edu

**Keywords:** pipelines, Structural Health Monitoring (SHM), Computational Fluid Dynamics (CFD), Genetic Algorithm (GA), corrosion

## Abstract

Internal corrosion is a major concern in ensuring the safety of transmission and gathering pipelines in Structural Health Monitoring (SHM). It usually requires numerous sensors deployed inside the piping system to comprehensively cover the locations with high corrosion rates. This study presents a hybrid modeling strategy using Computational Fluid Dynamics (CFD) and Genetic Algorithm (GA) to improve the sensor placement scheme for corrosion detection and monitoring. The essence of the proposed strategy harnesses the well-validated physical modeling capability of the CFD to simulate the oil-water two-phase flow and the stochastic searching ability of the GA to explore better solutions on a global level. The CFD-based corrosion rate prediction was validated through experimental results and further used to form the initial population for GA optimization. Importantly, fitness was defined by considering both sensing effectiveness and cost of sensor coverage. The hybrid modeling strategy was implemented through case studies, where three typical pipe fittings were used to demonstrate the applicability of the sensor layout design for corrosion detection in pipelines. The GA optimization results show high accuracy for sensor placement inside the pipelines. The best fitness of the U-shaped, upward-inclined, and downward-inclined pipes were 0.9415, 0.9064, and 0.9183, respectively. Upon this, the hybrid modeling strategy can provide a promising tool for the pipeline industry to design the practical placement.

## 1. Introduction

Pipelines conduct essential roles in transporting oil and gas across the nation, fueling the modern industry and way of life [1,2]. However, pipelines are claimed to suffer from internal corrosion due to the properties of the transported fluids, operating pressure, as well as the injected inhibitors [3]. According to the released data from the Pipeline and Hazardous Materials Safety Administration (PHMSA), internal corrosion accounts for approximately 60% of all corrosion-related incidents in transmission and gathering pipelines [4]. 

Internal corrosion poses many challenges to pipeline integrity, necessitating a comprehensive comprehension of its consequences. The gradual reduction in pipeline walls due to corrosion constitutes a primary concern, jeopardizing structural robustness and increasing susceptibility to leaks and ruptures. Such vulnerabilities, if left unchecked, can accumulate in catastrophic failures with severe implications for safety and environmental preservation. The financial toll of internal corrosion is equally substantial, encompassing the costs of repair, replacement, and lost productivity due to unscheduled downtime. Regulatory fines and penalties, coupled with the tarnishing of reputations, compound the financial ramifications. Furthermore, the accumulation of corrosion by-products can impede fluid flow, impacting operational efficiency and disrupting resource delivery to industries and households. By elucidating these challenges and potential risks, we underscore the pivotal significance of the proposed modeling strategy. This strategy’s ability to predict corrosion rates, identify vulnerable areas, and guide mitigation efforts resonates as a proactive solution to prevent corrosion-induced failures, ensuring safety, environmental protection, and financial stability within the pipeline industry.

The timely monitoring of internal corrosion in pipelines continues to pose significant challenges due to limited accessibility for regular inspection and maintenance. Moreover, internal corrosion often occurs discreetly and gradually at random locations along extensive pipeline networks, resulting in potentially severe consequences that may go unnoticed until significant damage has already occurred [5,6]. Also, additional environmental factors can exacerbate the impact of corrosion, leading to potentially catastrophic outcomes [7,8]. Thus, it is of paramount importance to locate corrosion events along the long-distance pipe system, in order to ultimately implement optimal corrosion control practices in Structural Health Monitoring (SHM) [9].

The fluid flow in pipelines is essentially a mixture of crude oil, water, and dissolved gases such as CO_2_ and H_2_S. It is well established that the distribution of water and oil inside the pipeline has a significant impact on the corrosion rate of the pipe wall [10]. To address the internal corrosion issues, oil-water two-phase flow is commonly employed to model the fluid behavior. On one hand, internal corrosion occurs when a free layer of the water phase comes in contact with the pipe wall. The flow patterns (i.e., water distribution) directly determine the phase for wetting on the inner wall of the pipeline. Considering that water and oil phases can exhibit different forms, such as emulsion (water-in-oil emulsions and oil-in-water emulsions), stratified, slug, or annular flow. It is necessary to model the fluid flow with consideration of the hydrodynamic parameters and geometric shape of the pipe that will impact the wetting conditions. On the other hand, the long distance of the pipeline network brings about difficulties in real-time corrosion detection and monitoring [11]. A balance between the sensing coverage for corrosion locations and the total expense of the sensor layout is hence required in engineering applications [12]. Based on this, a comprehensive strategy, which is capable of modeling oil and water phases with various flow characteristics and further predicting the corrosion rate accurately based on the flow-induced wall shear stress, is thus expected for the implementation of better corrosion practices.

Experimental measurements on pipeline internal corrosion are usually limited due to the uncertainty and difficulty in controlling the flow patterns [13]. Computational Fluid Dynamics (CFD) have been commonly used to simulate the flow behavior of a crude oil and water mixture, which significantly facilitates the parametric study on different flow characteristics. Hu et al. [14,15] carried out CFD analysis on the oil-water two-phase flow containing dissolved CO_2_. Importantly, a map of flow pattern was derived to demonstrate the potentials of corrosion under different conditionings with regard to water cut and flow velocity. The CFD analysis revealed that corrosion is prone to occur at the bottom part of the pipe when the flow pattern is stratified and dispersed. Particularly, when the water cut exceeds 80% and the flow velocity is greater than 1 m/s, the entire inner wall will be wetted with water. Additionally, the corrosion rates were also predicted using empirical models based on the shear stresses calculated from the CFD study. Results show good agreement with the actual measurements. Hassan et al. [13] performed a CFD study on the internal corrosion of oil-water two-phase flow in straight pipelines, addressing an emphasis on the distribution of water and the types of wetting (i.e., water wetting and oil wetting) that could result in corrosion at the bottom of the pipe wall. Zhang et al. [16] further investigated the bottom corrosion of the pipe wall in upward-inclined pipe fittings. The key parameters, such as the water cut, mixture velocity, oil viscosity, and inclination angle of the pipe, were analyzed. The simulation results show good coincidence with experimental measurements. Clearly, CFD simulation provides an efficient way to solve the complex fluid problems associated with sets of partial differential equations that describe the fluid flow. 

As stated above, CFD-based numerical analysis allows dealing with a large number of variables impacting the corrosion rate of the pipeline. On the other hand, a variety of commercially available corrosion sensors have been developed to monitor the corrosion process from causes to consequences using different sensing principles [16]. Numerous sensors serving different monitoring purposes are required to maintain the integrity of the corrosion inspection along pipelines, especially in large-scale infrastructures. Consequently, it brings about a large amount of investment in manufacturing and operating costs. Thus, the sensor optimization with regard to quantity and layout is hence essential to promise sensing efficiency and expense control. Upon this, data-driven approaches have been gaining increasing attention to solve complex (system) problems with typical characteristics in terms of high nonlinearity and stochasticity [16]. Sensor placement optimization based on the pipeline corrosion associated with the correlated parameters of the fluid flow can thus take advantage of the strong mapping ability of AI algorithms to find out an optimal solution, with adjusting the parameters during the prediction process. Among various algorithms, Genetic Algorithm (GA), due to its global searching ability without getting trapped in local minimal, is widely adopted in SHM fields. Cheng et al. [17] used GA to optimize the temperature and CO_2_ sensor placement for thermal comfort and indoor air quality monitoring under limited field measurements. The coverage checking and accuracy checking were conducted, respectively, to demonstrate the applicability of GA in sensor placement optimization. The developed GA program shows quick convergence and high fitness over approximately 40 iterations, indicating good efficiency for the optimization. Kim et al. [18] proposed an Adam-mutated GA approach to determine the optimal locations of sensors in the pipeline network for real-time monitoring. The evolutionary process in terms of mutation was further optimized by integrating a mutation operator to increase the robust ability to escape from the local minimal.

Motivated by the aforementioned sophisticated techniques, this study addresses efforts to integrate the strong nonlinear solving ability of CFD and the global stochastic searching ability of GA. The workflow of the hybrid modeling strategy using CFD and GA is exemplified in a step-by-step manner. The shear stresses of the pipe wall obtained from the CFD calculation are used to estimate the corrosion rate for different pipe fittings containing the oil-water two-phase flow. Importantly, the time steps divided in the CFD solving are recorded and thereafter used as the corresponding identification numbers of initial individuals in GA. The schemes of sensor placement for the U-shaped, upward-inclined, and downward-inclined pipes were optimized through the population evolutions of the GA to demonstrate the applicability of the developed strategy. 

## 2. Methodology of Hybrid Modeling for Sensor Placement Optimization

### 2.1. Framework of Hybrid Modeling Using CFD and GA

**Figure 1:**
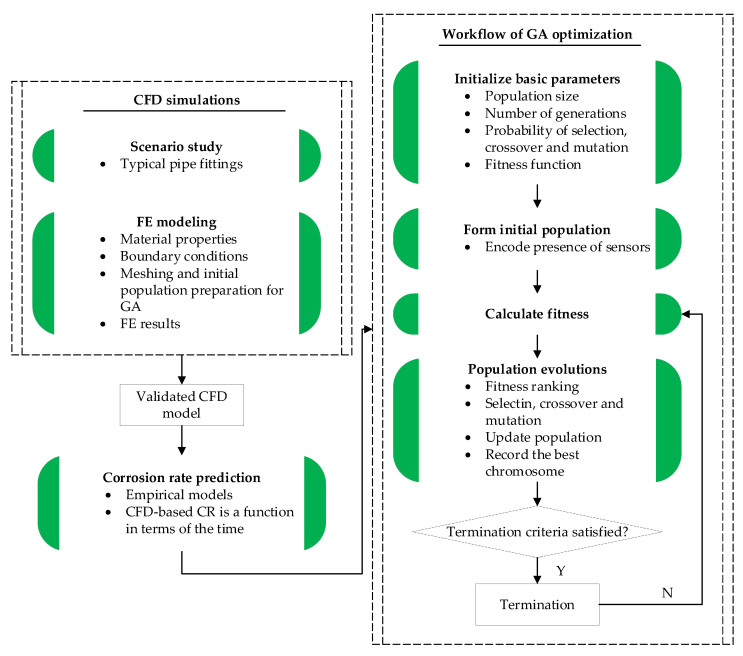
Flowchart of the proposed hybrid modeling strategy.

A systematic framework of the hybrid modeling strategy for internal corrosion sensor placement was proposed, as detailed in the flowchart Figure 1. Generally, it consists of two major modules: the CFD simulations that aim to capture the flow-induced shear stress distribution of pipe wall and the GA prediction to evolve the optimal solution for sensor placement along the pipeline until the target evolution is achieved. Importantly, the elements built in the CFD model constitute the population for the GA, thus creating a bridge of information exchange between the two modules. In the first module, as one of the most critical variables of corrosion rate, shear stress needs to be calculated. First, the CFD program ANSYS-Fluent is employed to simulate oil-water two-phase flow passing through pipelines. Considering that the bending segment of pipelines coming in an angle is prone to suffer from internal corrosion due to flow disturbance, the scenario study focuses on piping elbows with different bending degrees when modeling in the pre-processor workbench environment. Next, the geometry model is then assigned the corresponding material properties and has boundary conditions applied on it for exquisite Finite Element (FE) analysis. In this step, the element quantities will affect the calculation efficiency of the subsequent GA analysis. Therefore, it is necessary to finely mesh critical parts of the pipeline and coarsely mesh the general parts, so as to yield a good compromise between prediction accuracy and calculation efficiency. In addition, the standard turbulent k−ε model implanted in ANSYS-CFD fluid is used herein since the oil-water mixture exhibits a homogeneous emulsion without laminar characteristics, simulating the interaction between inner wall and fluid flow. Correspondingly, the FE results in terms of pipe wall shear stress distribution can be calculated, where the critical parts with higher shear stress require placement of sensors. Finally, the CFD simulations need to be validated with experimental results to prove its applicability in further study. Based on this, the empirical models applicable to different temperature and oil fraction can be used to forecast the corrosion rate of the pipe elements. In summary, the CFD simulation of the first module is to build a database containing critical information with regard to the corrosion rate and its corresponding position coordinates of the element, which can be used for the subsequent GA prediction. 

In the following, GA is launched to optimize the sensor placement. Basically, critical parameters in terms of population size, number of generations, probabilities of evolutionary events are initialized incipiently. Importantly, fitness is defined as a function that comprehensively considers the corrosion detection effectiveness for pipelines and the overall sensor cost, which is the core indicator for judging chromosome quality throughout population evolutions. As mentioned above, the elements are treated as chromosomes, of which the sensor presence is encoded. Note that chromosomes with larger fitness exhibit better performance; these superior chromosomes are more probable to allow evolutions in terms of crossover and mutations. Thereafter, better-performing chromosomes will substitute for those worse ones, forming a new population for the next evolution loop. After rounds of iterative evolution, until reaching the target evolution designated in advance, the best chromosome is selected and decoded to interpret the sensor array scheme. Toward the end, the optimal solution is thus expected to yield a good compromise between engineering accuracy and financial cost.

### 2.2. CFD Simulations

#### 2.2.1. Scenario Study for Different Pipe Fittings

The ANSYS-Fluent workbench using CFD solver was used in this study due to its wealth of forecasting ability for flow, velocity, temperature, and chemical concentrations for any part where flow occurs, as well as the high flexibility in research involving parametric and especially topological changes. It also brings about a significant reduction in computational cost compared to experimental work.

Piping layout design requires identifying and formulating the most appropriate and economical solutions and practices, taking into account the requirements for the pipe network to cross different elevations and bypass or traverse complex terrain. Upon this, piping elbows are the major pipe fittings that are frequently used for changing the direction of the flow in the piping system. However, the flow disturbance induced from the elbows, in turn, leads to severe damage to the pipeline itself. Changes in flow patterns can cause upset internal corrosion potential and rate. For example, low flow rates can lead to more opportunities of liquid or solid dropout and accumulation at low spots, whereas high flow rates can lead erosion due to abrasive or scouring effects of a transmission medium moving against the pipe wall. In addition, abnormal conditions such as the unintentional introduction of contaminants, might exacerbate previously slight internal corrosion potential [19]. Carbon steel elbows with a respective bending angle of 30°, 60°, and 90° were reported to suffer from internal corrosion in the literature [20,21]. In addition, a thickness of 6.4 mm of the pipe wall was used to represent the standard pipeline in the modeling.

**Figure 2:**
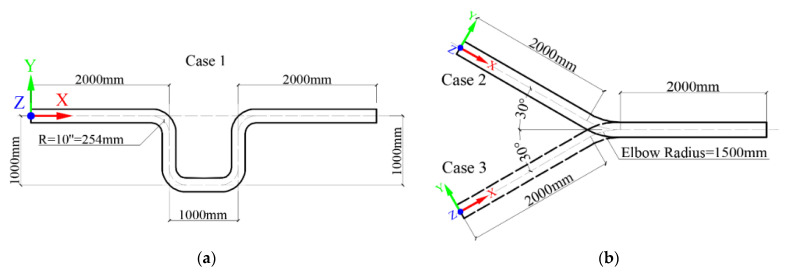
Typical pipe fittings: (**a**) U-shaped pipe and (**b**) upward-inclined and downward-inclined pipes.

Thus, the scenario study investigated three commonly used pipe fittings, viz. the U-shaped pipe (case 1) with a 90° bending angle, the upward-inclined (case 2), and downward-inclined (case 3) stream pipes with a 30° bending angle, respectively. Figure 2 shows the dimensions of the pipeline models considered in this study. The U-shaped geometry is primarily adopted in pipeline clusters and management stations, where side pipes and pipeline maintenance or rehabilitation equipment can be found. The geometry of upward-inclined and downward-inclined stream pipes are usually seen at terrains of mountain junctions and depressions of plains. In addition, considering that regions close to inlet and outlet boundaries display relatively higher stiffness than it should, the pipe length is suggested to have at least one time of the pipe’s diameter to avoid the end effect, according to Saint-Venant principle [22].

#### 2.2.2. Governing Equations

Numerical analysis of the oil-water two-phase flow was conducted using the Euler–Euler (EE) model. It describes both the fluid and particulate phases by employing transport equations within a consistent global coordinate system. In this approach, particle trajectories are not individually monitored in spacetime, rather, the focus lies on characterizing the distribution of properties within the particle phase (such as velocity, size, composition, etc.). This model finds application in simulating two-phase scenarios, wherein two liquid substances are regarded as mutually interpenetrating continua. The mixture model is built based on the average mass and momentum equations for each phase, which calculates the volume fraction of the second phase and the velocity slip at the water–oil interface by solving the governing equations of the mixture [23]. The water and oil phases share the same pressure field. In this work, it is assumed that there is no mass transfer between phases. For an incompressible fluid, the continuity and momentum equations are written as follows:

(1) $$\nabla \cdot \left(\rho_{k}\alpha_{k}\vec{v_{k}}\right)=0$$

(2) $$\nabla \cdot \left(\rho_{k}\alpha_{k}\vec{v_{k}}\vec{v_{k}}\right)=-\alpha_{k}\nabla P+\rho_{k}\alpha_{k}\vec{g}+\nabla \left[\alpha_{k}\mu_{k}\left(\nabla v_{k}+{\left(\nabla \vec{v_{k}}\right)}^{T}\right)\right]+\vec{F_{k}}$$

where ρ, α, and $\mu_{k}$ are density (kg/m^3^), volume fraction, and effective viscosity (Pa·s). In the momentum equation, the terms of the right-hand side stand for the pressure gradient (Pa/m), stress (N/m^2^), gravity (m/s^2^), and the interfacial forces (N) applied on the phase k. The secondary phase p can be solved using the same equations. 

#### 2.2.3. Turbulence Model

There are several turbulent models implanted in the ANSYS-CFD program to simulate fluid flow in pipelines, including the algebraic eddy models [24], one-equation models such as the Baldwin–Barth [25] and Spalart–Allmaras models [26], as well as the two-equation models [27]. Among them, the K-epsilon (*k-ε*) turbulence model has been extensively used for numerous applications and generally performs quite well for practical engineering applications. It yields robust reliability under free-shear flow conditions. It falls under the category of two-equation turbulence models and is specifically designed to simulate turbulent flows in a variety of engineering applications [28]. The model’s name refers to the two transport equations it utilizes, one for turbulent kinetic energy (k) and another for turbulent dissipation rate (ε). In the *k-ε* turbulence model, the turbulent kinetic energy (k) represents the energy associated with the fluctuations in velocity in turbulent flows, while the turbulent dissipation rate (ε) quantifies the rate at which turbulent kinetic energy is dissipated into heat due to viscous effects. This model is particularly useful for simulating turbulent flows where the Reynolds number is sufficiently high, making the direct simulation of all turbulence scales computationally expensive.

In this work, the standard *k-ε* model uses two transport equations to describe the turbulence flow, the transport of the turbulent kinetic energy (k), and the turbulent energy dissipation (ε), as illustrated in Equations (3) and (4), respectively:

(3) $$\frac{\partial \left(\rho k\right)}{\partial t}+\frac{\partial \left(\rho ku_{i}\right)}{\partial x_{i}}=\frac{\partial }{\partial x_{j}}\left[\frac{\mu_{t}}{\sigma_{k}}\frac{\partial k}{\partial x_{j}}\right]+2\mu_{t}E_{ij}E_{ij}-\rho \varepsilon$$

(4) $$\frac{\partial \left(\rho \varepsilon \right)}{\partial t}+\frac{\partial \left(\rho \varepsilon u_{i}\right)}{\partial x_{i}}=\frac{\partial }{\partial x_{j}}\left[\frac{\mu_{t}}{\sigma_{\varepsilon }}\frac{\partial \varepsilon }{\partial x_{j}}\right]+C_{1\varepsilon }\frac{\varepsilon }{k}2\mu_{t}E_{ij}E_{ij}-C_{2\varepsilon }\rho \frac{\varepsilon^{2}}{k}$$

where $u_{i}$ is the velocity components in corresponding directions; $E_{ij}$ is the deformation rate; and $\mu_{t}$ is the eddy viscosity, which can be calculated using Equation (5). The application of consistent SI units pertains to the Partial Differential Equations.

(5) $$\mu_{t}=\rho C_{u}\frac{k^{2}}{\varepsilon }$$

Specifically, the constants used in the CFD simulation were set to be $C_{u}$ = 0.09, $\sigma_{k}$ = 1.0, $\sigma_{\varepsilon }$ = 1.3, $C_{1\varepsilon }$ = 1.44, and $C_{2\varepsilon }$ = 1.92.

#### 2.2.4. Boundary Conditions

For the two oil and water phases, the inlet boundary conditions use velocity boundary to illustrate the oil and water entering the pipe. The inlet velocity of the mixture and water cut can be defined to meet different operating conditions. In addition, the turbulent intensity and hydraulic diameter are also specified at the velocity inlets. Especially, oil was considered as a primary phase. The pressure boundary conditions were applied at the outlets with the gage pressure of zero. In addition, the non-slip boundary conditions were used for the pipe wall. The impact of the wetting surface was applied to the wall through adjusting the contact angle, where the steel pipe segment was regarded as a hydrophilic surface.

#### 2.2.5. Meshing Strategy

**Figure 3:**
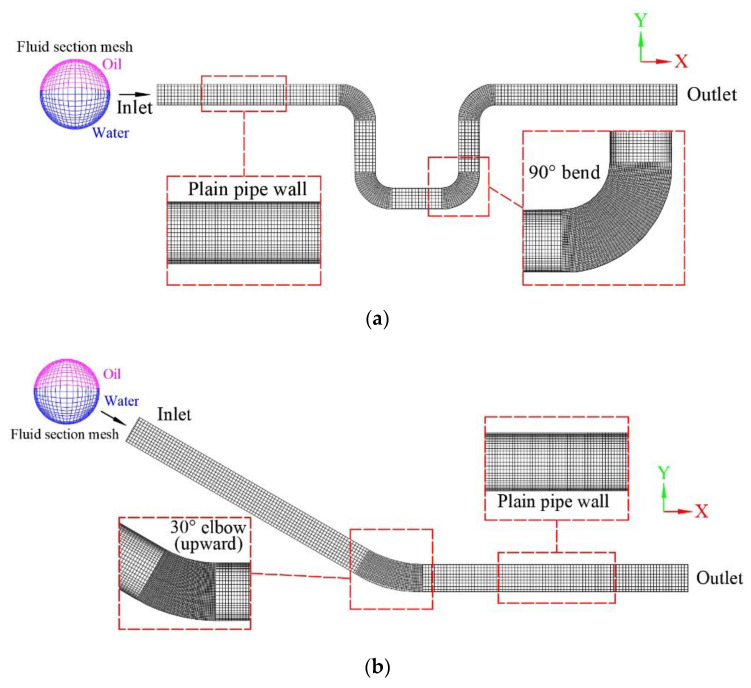
Meshing for typical pipe fittings: (**a**) U-shaped pipe and (**b**) inclined pipe.

In hybrid modeling, the total number of elements determines the population size of the GA, which in turn affects the prediction and optimization of the GA. Thus, the meshing needs to yield a good compromise between simulation accuracy and computational cost. Furthermore, the selection of element shape also has a significant impact on the convergence efficiency of nonlinear iterative calculations. In general, the meshing strategy is to use hexahedrons due to its capability to achieve a high quality solution with a fewer number of elements [29]. On the other hand, the elements adjacent to the bending locations need to be finely meshed with smaller grid cell size compared to the rest of the pipe, as illustrated in Figure 3. More importantly, a reasonable meshing strategy ensures a limited computational cost of the subsequent GA optimization.

### 2.3. Corrosion Rate Predictions

The corrosion rate (CR) of oil-water two-phase flow in pipelines was predicted using the empirical expression that is applicable to the scenarios where the volume fraction of oil is less than 60% and the temperature is below 60 °C [30]:

(6) $$CR=kp^{c}\tau^{b}$$

where CR is corrosion rate (mm/yr), k is the coefficient with a value of 15.5 ± 0.5, and *p* is the carbon dioxide partial pressure (MPa); *c* is the exponent with a value of 0.83 ± 0.07, τ is wall shear stress (N/m^2^), and b is the exponent of wall shear stress with a value of 0.1. It is worth mentioning that the corrosion rate of materials can be significantly impacted by the partial pressure of carbon dioxide (CO_2_) in the surrounding environment. When CO_2_ dissolves in water, it forms carbonic acid, leading to a decrease in pH. The resulting acidic conditions can accelerate the corrosion process by promoting the dissolution of metals. Higher CO_2_ partial pressure results in greater concentrations of dissolved CO_2_ and thus a more pronounced corrosive effect. This phenomenon is particularly relevant in industries involving pipelines, marine structures, and chemical processes, where CO_2_-rich environments are common. The formation of carbonic acid increases the availability of hydrogen ions, which can participate in electrochemical reactions that facilitate metal dissolution. Moreover, the localized decrease in pH due to carbonic acid can lead to the initiation and propagation of localized forms of corrosion, such as pitting. Therefore, understanding the impact of carbon dioxide partial pressure on corrosion is crucial for effectively managing and mitigating corrosion-related issues in various industrial settings. Particularly, carbon dioxide partial pressure was found to have no effect on the corrosion rate when the oil concentration was above 80% [30]. 

When the volume fraction of oil is greater than 70%, the corrosion rate can be calculated using the following [31]:

(7) $$CR=31.15{\left(\frac{\Delta P}{L}\right)}^{0.3}v^{1.6}p^{0.8}e^{\left(-\frac{2671}{T}\right)}$$

where $\frac{\Delta P}{L}$ is pressure gradient (N/m^3^); v is the water cut; and T is the temperature (*K*). A higher water cut in pipelines, indicating increased water content relative to oil, can intensify internal corrosion rates. Water promotes the formation of acidic environments, accelerating metal dissolution. The enhanced presence of electrolytes in water accelerates the electrochemical corrosion process, posing a heightened risk of corrosion-related failures.

The CFD analysis is divided into a set of time steps from 0 to *N*, in which the shear stress of element *j* at time *i* can be obtained. The corrosion rate is essentially a function in terms of the time *t*. The corrosion rate of all the elements at a certain time step can be estimated based on Equations (6) and (7), constituting the initial population of GA that can be used for the optimization of sensor placements.

### 2.4. GA Modeling and Optimization for Sensor Placement

#### 2.4.1. CFD-Based Architecture and Working Principle of GA

**Figure 4:**
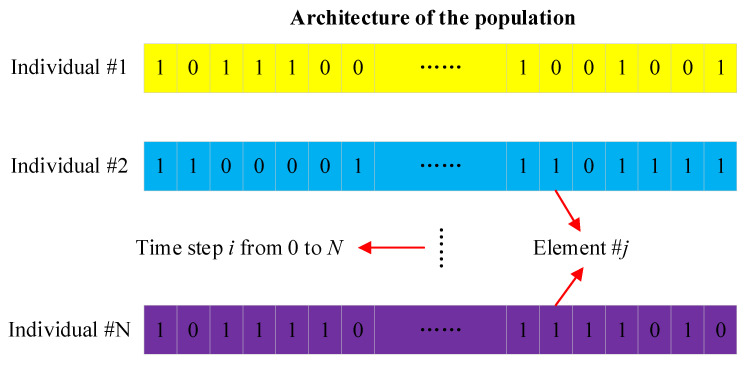
CFD-based architecture of GA.

The first step of GA optimization for sensor placement is to determine the architecture and chromosome constituents. As discussed in Section 2.1, the number of elements will govern the length of the individuals in the population, thus affecting the computational cost of GA. By applying the aforementioned meshing strategy, the number of elements is limited to a reasonable range, and the sensor-related information of all the elements is stored in the genes of the individuals. Figure 4 details the architecture of the population of GA. Each individual represents a scheme of the sensor placement, and these individuals constitute a population. The binary encoding strategy is adopted to implement an individual in a 0 and 1 sequence format. For example, encoding with 1 indicates the presence of a sensor associated with an element, while encoding with 0 indicates the absence of a sensor. Thus, the gene sequences in an individual stipulate the layout of the sensor array for all the elements. Different individuals represent different layouts, ranging—at the time step *i*—from 0 to *N* based on the CFD analysis. Furthermore, all the individuals can be evaluated based on a criterion, which is essentially defined as fitness and will be discussed in the following section. 

**Figure 5:**
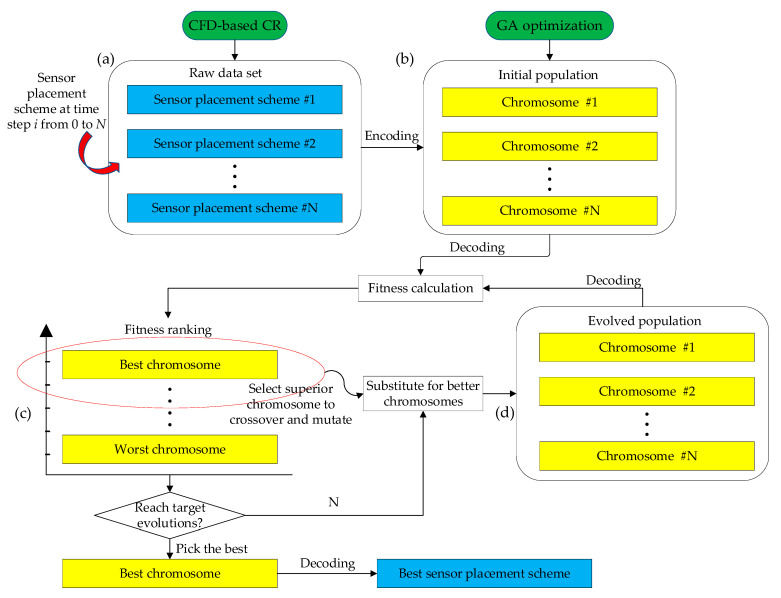
Working principle of GA.

Figure 5 demonstrates the working principle of the GA. Generally, it benefits from the global stochastic searching ability to comprehensively consider the corrosion risk of pipelines and the overall cost of the sensor layout plan. The essence of the GA is to utilize the principle of survival of the fittest in biology, seeking optimal solutions on a global level with evolutionary operations in terms of selection, crossover, and mutation. The mutated chromosome will be screened to form a new population by calculating the fitness. The evolution process is repeated until an ideal solution is finally obtained [32,33]. First, the raw data set needs to be preprocessed and classified in advance. Individuals containing information of the sensor layout plan for all the pipeline element data are arranged in the order of the time step *i* from 0 to *N*, constituting a set of schemes for sensor placement, as shown in Figure 5a. These individuals are encoded to form a number of chromosomes, as shown in Figure 5b. To evaluate the chromosome performance, all the chromosomes need to be decoded to calculate their respective fitness. Note that chromosomes with larger fitness exhibit better performance, and these superior chromosomes are more probable to allow evolutions in terms of crossover and mutations, as shown in Figure 5c. Thereafter, better-performing chromosomes will substitute for those worse ones, forming a new population for the next evolution loop, as shown in Figure 5d. After rounds of iterative evolution, until reaching the target evolution designated in advance, the best chromosome is selected and decoded to interpret the sensor array scheme. The optimal solution is thus expected to yield a good compromise between engineering accuracy and financial cost.

#### 2.4.2. Time Complexity for Modeling Decision Making

The hybrid modeling of CFD and GA involves correlated parameters contributing to the final prediction accuracy and time efficiency. For example, the number of elements in the FE model determines the length of the chromosome in GA; the number of time steps defined in the FE model determines the population size in GA. These correlated parameters have a significant impact on the fitness accuracy of the GA optimization. Therefore, the hybrid modeling strategy requires an indicator to comprehensively cover these variables, in order to demonstrate the feasibility and efficiency in practical engineering projects of sensor placement. Based on this, the time complexity was used herein, facilitating the evaluation of the hybrid modeling decision making. Generally, it is dependent on the representation of chromosomes, population size, number of generations, probabilities of crossover, and mutation. Consequently, the time complexity can be calculated using the following equation [34]:

(8) $$T=G(S\times \log\left(S\right)+\left(2\times p_{c}\times S\times n\right)+\left(p_{m}\times S\times n\right)$$

where *G* is the maximum number of generations; *S* is the population size (i.e., number of time steps); *p_c_* is the probability of crossover; *p_m_* is the probability of mutation; and *n* is the chromosome length (i.e., number of elements in the FE model). Clearly, the population size has a huge impact on the time complexity, leading to significant changes. Furthermore, noting the multiplicative relationship of the population size and chromosome length indicates that these two parameters will have an exponential amplification effect on the time complexity. Therefore, it is suggested to strictly follow the meshing strategy stipulated in Section 2.2.3, which applies fine mesh in critical parts and coarse mesh in the remaining. Importantly, the final parameter settings will be evaluated based on the fitness to meet the requirement of the sensor optimization.

#### 2.4.3. Parameters Settings of GA

Key parameters of the GA, such as the population size, the maximum number of offspring, and the probability evolutionary events, affect the optimization results and convergent efficiency [35]. Especially for the hybrid modeling in this work, some parameters are correlated. For example, the meshing of the FE model will affect the length of the chromosome, in which the information about the sensor presence is stored in sequence of the element numbers. In addition, the time steps defined in the CFD solver will directly determine the population size, ultimately influencing the computation time for the GA evolutions. Basically, the more chromosomes that the population involves, the more options there are to conduct evolutionary operations to finally find more superior offspring. However, this simultaneously increases computational cost and leads to a long time of evolutions. Thus, it is necessary to achieve a good balance between accuracy and efficiency. The maximum number of generations represents the total iterations, which is dependent on the fitness variations. When the fitness does not significantly improve with the increase in the evolutions, the value of the demarcation point is a reasonable solution to meet engineering accuracy. In most cases, the probability of selection is usually set to a larger value (greater than 80%), so that most chromosomes will have a higher probability of being allowed to evolve; the mutation probability is usually set to a smaller value to avoid anomalous solutions that deviate too much from the requirements of the project; and the probability of crossover is set in between.

#### 2.4.4. Crossover and Mutation

**Figure 6:**
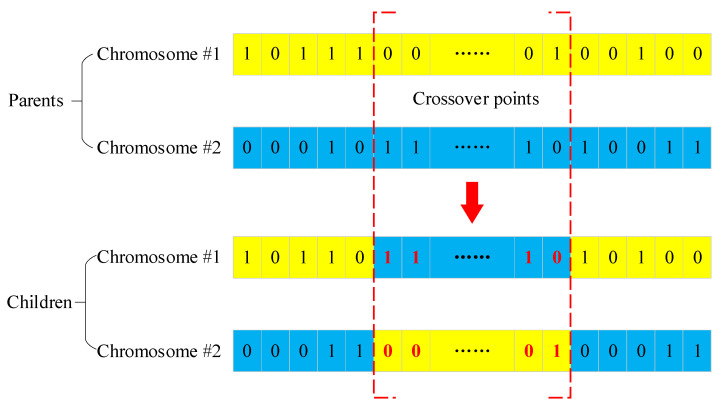
Two-point crossover mechanism between two chromosomes.

The GA aims to explore better solutions through successive population evolutions, where operations with regard to crossover and mutation among chromosomes are of paramount importance to avoid falling into local minima, thus ultimately picking up the optimal solution on a global level. In this work, a crossover was implemented with the randomly switching chromosome section to mimic the behavior of gene exchange and recombination between chromosomes [32]. Generally, crossover can be categorized into three major types: one-point crossover, multi-point crossover, and uniform crossover [36]. In one-point crossover, a crossover point is picked randomly from the parent chromosomes, and the tails of the two chromosomes are swapped to generate the children. Multi-point crossover is essentially a generalization of the one-point crossover wherein multiple points are picked randomly, and the alternating segments are swapped to generate new children. Unlike these two types of crossovers, the uniform crossover, typically, is more complicated. The chromosome is not divided into segments. Instead, each gene is treated separately to decide the possibility of its inheritance for the children chromosome. Consequently, numerous computing resources are thus required to simulate the regeneration mechanism during the repetition of decision making. Considering the huge initial population obtained based on the CFD time steps, a two-point crossover was selected herein, to yield a compromise between engineering accuracy and computing time efficiency. Figure 6 demonstrates the two-point crossover mechanism. Clearly, the children (offspring) inherit genetic information through crossover between the two chromosomes in the parents.

**Figure 7:**
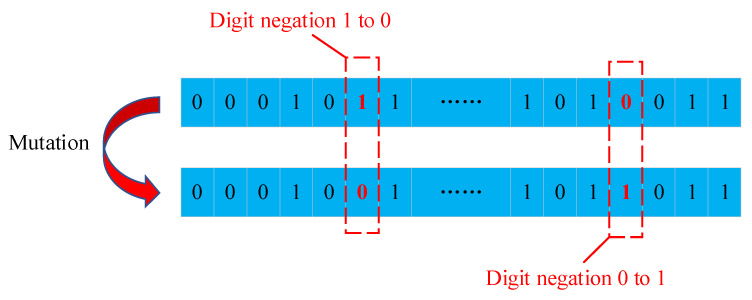
Mutation of a chromosome.

In addition to crossover, mutation is an occasional reversion of the gene on a chromosome to keep evolving into more superior ones, thus maintaining the diversity of the population. Importantly, mutation in the GA is an attempt to avoid local minima, where the chromosomes in a population exhibit high similarity among each other, thus leading to a slow, or even stopping, convergence to global optimum. The dominating factor of the mutation is the mutation probability of the gene in a chromosome to change, resulting in a complete new sequence array [32]. Figure 7 demonstrates the mutation of a chromosome. Since this work adopts a binary format that contains only 0 and 1 for chromosomes, the mutation was implemented by triggering the digit negation.

#### 2.4.5. Fitness Function

Fitness is the most critical indicator of the GA to evaluate the quality of chromosomes in a population and promote the evolutions of the population based on the theory of survival of the fittest. For sensor placement, two primary factors need to be considered. First, the sensor layout plan requires that that there should be sensors at locations where the corrosion rate is higher than the allowable value, to ensure timely detecting and monitoring. Secondly, the layout plan should yield a reasonable expense that the project budget can accept. Based on this, the optimization aims to find a scheme with a high sensing effectiveness and a low cost. Critical locations with high corrosion risks need to be highlighted while considering the overall cost. Therefore, the fitness function of a particular potential sensor location (*x*, *y*, and *z*) on the pipeline at the time *i* is specifically designed for pipeline corrosion sensor placements by considering these two primary factors, including the corrosion rate at a certain location and cost:

(9) $$f\left(i,j\right)=max\left[w\times a\left(i,j\right)+\left(1-w\right)\times c\left(j\right)\right]$$

where *w* is the weight factor, *a* is the corrosion rate function, and *c* is the cost function, respectively. *j* is the sensor location identification number, and the sum of *j* will be the maximum number of sensors *n*. The function of the corrosion rate *a*$\left(i,j\right)$ is defined as 1 − *e*$\left(i,j\right)$, where *e*$\left(i,j\right)$ is the variance function that describes the difference between the maximum corrosion rate and the estimated corrosion rate at the current sensor location. The rationale behind subtracting the estimated corrosion rate from the maximum corrosion rate lies in optimizing sensor placement. By minimizing the difference *e*$\left(i,j\right)$ between the maximum and estimated corrosion rates, the algorithm seeks to identify sensor locations where the predicted corrosion rate closely aligns with the worst-case scenario. This strategy prioritizes accurate detection of critical corrosion-prone areas, minimizing the risk of unexpected failures and ensuring effective resource allocation. The weighted combination of the corrosion rate function and cost function further allows for a balanced decision-making approach, considering both corrosion severity and associated costs. It can be expressed as follows:

(10) $$a\left(i,j\right)=1-e\left(i,j\right)=1-\sqrt{{\left(\frac{F_{actual,i}-F_{i}\left(j\right)}{F_{actual,i}}\right)}^{2}}$$

Thus, as the variance tends to be 0, the corrosion rates at these locations are maximum corrosion rates that can be tolerated, and the corrosion rate function will be equal to 1 and vice versa. $F_{i}\left(j\right)$ is the estimated corrosion rate from simulation tools such as Computational Fluid Dynamics (CFD) at these sensor locations. $F_{actual,i}$ is the actual maximum allowable corrosion rate at certain locations, *j* is the sensor identification number that can be described as the data set location, and the sum of *j* equals the total data set. 

The second part of the optimization function in Equation (9) is the cost function, which is given as follows:

(11) $$c\left(j\right)=\frac{1}{\sum_{1}^{N}j\times b}$$

where ∑j is the sum of the sensors based on the sensor location *j*, and b is the unit price of the corrosion sensor. Thus, the maximum c$\left(j\right)$ is 1 when no sensors are placed, and the minimum c$\left(j\right)$ is 0 when all the locations have been placed with sensors.

After getting these critical positions of high corrosion risks with the determining corrosion rates, 70% of the obtained data from the simulation were used to train the GA algorithm, and 30% of the data were used for validation. The unit cost *b* is USD 50.00, and the weight factor *w* is set to 0.5 to iterate the fitness function. The GA will provide chromosome encoding, mutation, crossover operation, and sensitivity analysis on the internal corrosion sensor placements.

## 3. Implementation of Hybrid Modeling for Sensor Placement Optimization

### 3.1. Critical Operating Parameters 

The fluid flow in pipelines is considered as an oil-water two-phase flow [37]. Internal corrosion tends to occur at the water-wetting of the pipe wall. The flow velocity and oil content are significant factors contributing to the flow patterns for both straight and inclined pipelines [38,39]. The water phase tends to accumulate at the bottom of the pipes when the flow velocity is below 0.5 m/s. Oil-in-water and water-in-oil emulsions are formed at higher flow rate conditions, where water is observed to touch both the top and bottom surfaces of pipelines. Especially, the water phase starts to show up in every level inside the pipelines when the water fraction exceeds 20% in weight. Otherwise, the oil dominates the fluid flow, preventing the upper part of the pipe from being water-wetted [14,15]. Based on this, the uniform water–oil emulsion was expected for the present work by adopting an equal fraction of water and oil in weight. Meanwhile, the flow velocity was set to be greater than 0.5 m/s, to trigger the occurrence of internal corrosion in the upper part of the pipe wall. 

Considering the prevailing operational conditions, it is pertinent to acknowledge that the temperature range of the natural gas within pipelines spans from 100 °F to 120 °F (37.78 to 48.89 °C) as reported in [40]. In this study, a pragmatic approach is taken to select the baseline temperature within the system at 40 °C since the actual temperature distribution along the pipeline is not available. This choice is made without compromising the broader applicability of the study, which is also consistent with the typical pipeline gas specifications. In practice, if the temperature distribution is available, the accuracy of the sensor placement can be further improved by providing the actual temperature profiles along the pipeline. For the water phase in reality, CO_2_ is dissolved in water [14,15]. The content of CO_2_ in the fluid carried via pipelines is not saturated. In the present work, 0.02 M of CO_2_ was assumed to be dissolved in water to approximate the normal condition in petroleum pipelines. Meanwhile, 0.02 M NaCl and 0.02 M NaHCO_3_ solutions were added to simulate the brine properties of the fluid under high corrosion risk [41]. Table 1 details the hydrodynamic properties of oil and water used for CFD simulations. The Conoco LVT 200 oil was selected as the oil phase. The dissolution of CO_2_ slightly changes the vapor pressure to 7.3 kPa [30,37]. In addition, the pipe wall used ASTM A53 carbon steel, which is extensively applied in ordinary steam, water, and gas pipelines. 

As demonstrated in Figure 2a,b, the left end of the pipe is the fluid inlet for all three cases, and the right end of the pipe is the fluid outlet. The input velocity of the fluid is consistent with the front section of the pipes. For example, the flow injects horizontally in the U-shaped pipe, which is parallel to the X-axis shown in Figure 2a. For the flow inside the pipes, velocity and pressure boundary conditions were applied at the inlet and the outlet, respectively. In addition, the fluid at the pipe wall was considered as stagnant, and the wall boundary condition was applied at the fluid layer in contact with the wall [42,43]. In addition, the mass transfer and the phase change were negligible in this simulation study. The inlet velocity of the fluid was assigned to 1.0 m/s. The outlets of the pipes were defined as free ends, indicating that the pressure of the fluid at the outlet surface was set to zero. The convergence criterion utilizes the residual value of variables in the calculation. And the simulation progress is transient with 1000 time steps, a 0.01 step size, and 100 iterations at each step.

### 3.2. Grid Independent Study

**Figure 8:**
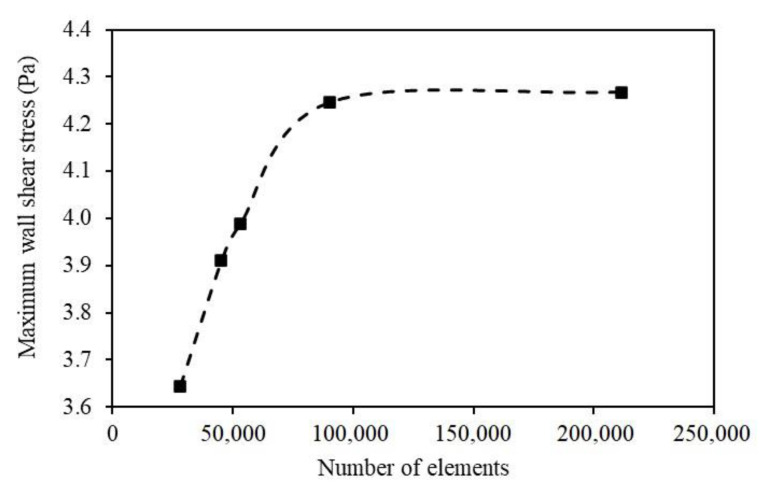
Maximum shear stresses under different meshing sizes.

The U-shaped, upward-inclined, and downward-inclined pipes were created using Autodesk Revit to demonstrate the commonly adopted fittings in pipelines. The geometry models were exported in SAT format so that the ANSYS-CFD could read the geometric information for further modeling and analysis. The meshing operation followed the meshing strategy stated above. Refined mesh was applied at the bending section, where a smaller element size was defined compared with the straight pipe section. In order to yield the appropriate element size and simulation accuracy, a grid-independent study was conducted with the following procedures: first, an empirical coarse size was set for the straight pipe, then the size was decreased by 0.005 m for each trial until the maximum shear stress could be accurately calculated. For example, as shown in Figure 8, the maximum shear stress increases rapidly with the increase in the number of elements, indicating that the mesh optimization has a significant effect on improving the calculation accuracy. The plateau shows that continuously increasing the number of elements (after approximately 100,000) has little effect on the improvement of calculation accuracy. Table 2 details the trials for the determination of element size. In meshing trial No. 2 and No. 3, the maximum shear stress was 4.2455 Pa and 4.2670 Pa with a total element number of 90,288 and 211,580, respectively. It can be seen that the accuracy of the maximum shear stress increased approximately 0.5% when the total element number increased approximately 100%. Consequently, a significant increase in element numbers results in great computational burden. Therefore, the meshing trial No. 2 yields a good balance between accuracy and computational cost. The appropriate meshing size was determined to be 0.015 m for the elbow section and 0.020 m for the remaining. The final optimal numbers of elements were found to be 13,192, 90,288, and 90,288 for the U-shaped, upward-inclined, and downward-inclined pipes, respectively.

### 3.3. Preliminary Estimation of Time Complexity

Table 3 details the preliminary estimation of the time complexity based on Equation (6). The probabilities of crossover and mutation were set to be 0.7 and 0.1, respectively. The length chromosome is equal to the total number of the elements in the CFD model. Thus, the lengths of chromosomes for case 1, case 2, and case 3 were 13,192, 8841, and 8841, respectively. The population size is equal to the total number of time steps in the CFD analysis. As suggested in Section 2.4.3, after a number of trials, time steps with a value of 1000 were found to yield a smooth convergence for the CFD solver during the iteration process. Meanwhile, the population size of 1000 was expected to maintain a good diversity of the population. Consequently, the time complexity for all the cases can be determined. It can be seen that the time complexity of the U-shaped pipe is much larger than that of the inclined pipe due to the meshing density of the curved segments. 

### 3.4. Validation for CFD-Based Corrosion Rate Prediction

The shear stresses calculated with the CFD simulation were used to predict the corrosion rates as demonstrated in Section 2.3. The experimental results conducted by Kanwar [30] were used to validate the accuracy and reliability of the CFD simulations. The test conditions of the Conoco LVT200 oil-water two-phase flow were summarized in Table 1. In Kanwar’s test, the carbon dioxide partial pressures with the value of 0.27, 0.45, and 0.79 MPa were used to investigate their effect on the corrosion rate. It revealed that the corrosion rate did not change when the carbon dioxide partial pressure increased from 0.27 to 0.79 MPa, as listed in Table 4.

**Figure 9:**
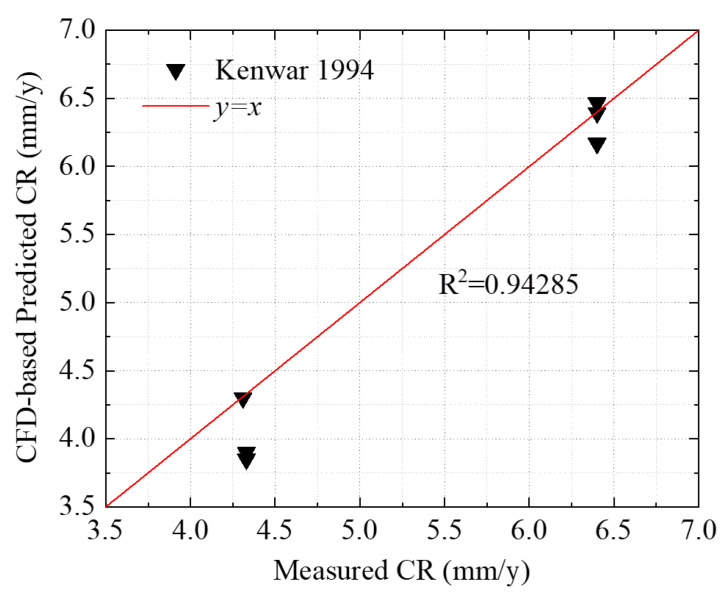
CFD-based predicted corrosion rates compared with experimental results.

Figure 9 shows the CFD-based predicted corrosion rates and measured corrosion rates. Equations (6) and (7) were used to predict the corrosion rate when the oil fraction was less than or greater than 80%, respectively. It can be seen that the *R^2^* (Pearson’s correlation coefficient R-value) was approximately equal to 0.94, indicating the CFD-based model fit well with experimental results. Therefore, the CFD model is capable of providing accurate data to the GA prediction and optimization for sensor placement.

## 4. Results and Discussion

### 4.1. GA Optimization for Sensor Placement

The internal corrosion rates can be calculated based on Equations (6) and (7). The total data points for the U-shaped, upward-inclined, and downward-inclined pipes are 13,192, 8841, and 8841, respectively. Table 5 lists the top 10 corrosion rates for each case and the corresponding coordinates.

**Figure 10:**
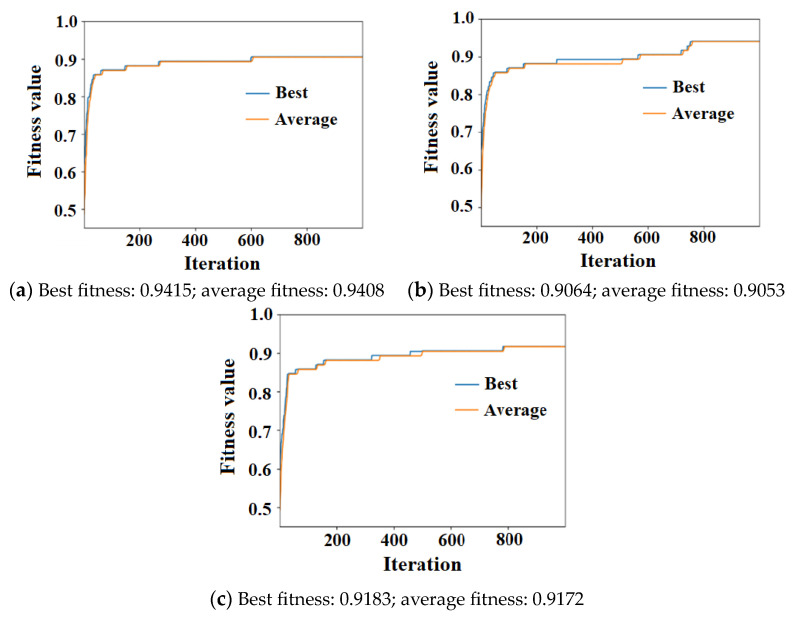
Fitness changes over number of iterations of (**a**) U-shaped pipe, (**b**) upward-inclined pipe, and (**c**) downward-inclined pipe.

Figure 10a–c show the fitness changes with the number of iterations for the U-shaped, upward-inclined, and downward-inclined pipe models, respectively. Clearly, the fitness first increased rapidly as the number of iterations increased. After a certain number of iterations, the plateau indicates that continuously increasing the number of iterations did not have apparent improvement for the fitness. In Figure 10a, the appropriate number of iterations of the U-shaped pipe was around 600, and the corresponding best fitness and average fitness were 0.9415 and 0.9408, respectively. Figure 10b,c show similar trends. For the upward-inclined pipe, the best fitness and average fitness were 0.9064 and 0.9053, respectively. For the downward-inclined pipe, the best fitness and average fitness were 0.9183 and 0.9172, respectively.

After the GA optimization, the individual with the highest fitness was extracted as the output. Since the chromosome is binary encoded, it is necessary to decode the chromosomes for the three cases. The sensor presence of all the elements can be determined through whether the sensor is placed (denoted as the value of 1) or not placed (denoted as the value of 0) in a bunch of locations along the pipes. From the GA results, a total of six, nine, and eight locations were designated as sensor deployment spots for the U-shaped, upward-inclined, and downward-inclined pipes, respectively. Table 6 shows the coordinates of the sensor locations with corresponding CRs. 

**Figure 11:**
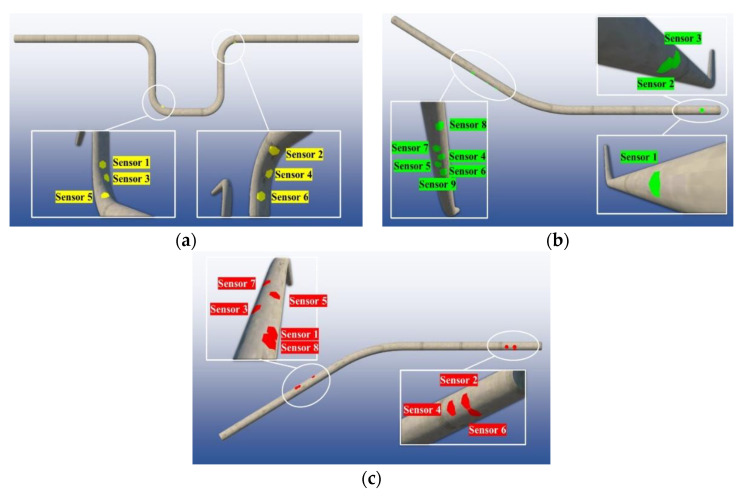
GA-optimized sensor placement plans: (**a**) case 1: 6 sensors, (**b**) case 2: 9 sensors, and (**c**) case 3: 8 sensors.

Figure 11 shows the sensor deployment plans for the three cases. In Figure 11a, there were a total of six sensors deployed at the bending locations. At the lower elbow of the U-shape pipe, three grouped sensors were suggested on the upper surface of the anterior elbow. While at the upper elbow, the same sensor arrangement was suggested at the lower surface. The final sensor design scheme of the U-shaped pipe is illustrated with yellow-highlighted marks. The sensor placement of the upward-inclined pipe with specified coordinators is shown in Figure 11b. There were a total of nine sensors, six of which were suggested to be deployed in the mid-section of the flush-out pipe segment on the lower surface, and three sensors were suggested near the inlet area of the upward pipe. The final sensor design scheme of the U-shaped pipe is illustrated with green-highlighted marks. For the downward-inclined pipe, a total of eight locations are highlighted with a red color. As seen in Figure 11c, the majority of the sensors were placed in the mid-range of the flush out pipe section on the upper surface. And three sensors were intended to be placed at the inlet area. 

On the other hand, the sensor placement plans without considering GA optimization were used herein to demonstrate the essence of the hybrid modeling strategy that utilizes the fitness function to strike a balance between efficiency and cost. Considering the wall thickness was set to be 6.4 mm, it is reasonable to conservatively control the maximum allowable corrosion rate within 5.00 mm/yr. Table 7 shows the total costs of sensor placement plans using different methods by the two scenarios with and without deploying the developed GA algorithm.

### 4.2. Impact of Fluid Velocity on Sensor Placement

**Figure 12:**
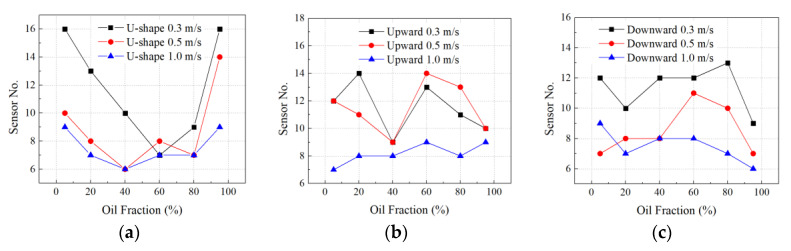
GA-optimized sensor quantity results of (**a**) U-shape, (**b**) upward-, and (**c**) downward-inclined pipes.

The GA optimization for sensor placement was applied to all the scenarios with multiple oil fraction and fluid velocity for U-shaped, upward, and downward pipes. The optimized sensor quantities under the scenarios of oil fraction: 5%, 20%, 40%, 60%, 80%, and 95%, with 0.3 m/s, 0.5 m/s, and 1.0 m/s fluid velocities, are concluded in Figure 12. It is shown that when the fluid velocity increased, the overall sensor quantity decreased. It is because higher fluid velocity led to the harsher stress concentration at pipeline elbows and turning segments, which demanded the sensors to focus on these critical locations instead of a broader spread along the pipe walls. The U-shaped pipe demands more sensors than the upward- and downward-inclined pipes. The maximum number of sensors of U-shaped pipes is 16, compared with that of the upward- and downward-inclined pipes, which are 14 and 13, respectively.

### 4.3. Impact of Oil Fraction on Sensor Placement

As shown in Figure 12, six levels of oil fraction are investigated for sensor location optimization. In Figure 12a, it is shown that when oil fraction is between 40% to 60%, the sensor number is optimized to as low as six. It is revealed that the equal oil-water fraction condition has the simplest sensor placement plan where the most significant spots are selected. When the fluid is either oil or water dominating, the sensor quantity goes up for a more complex sensor location distribution. 

Under inclined pipe conditions, the sensor quantity shows unostentatious change. In upward-inclined scenarios (Figure 12b), the sensor number fluctuates between 5 and 14. While in the downward-inclined scenarios (Figure 12c), the sensor number shows a slight drop tendency, with the minimum six sensor locations optimized. It is demonstrated that the complexity of the sensor deployment locations has a weak contribution to the pipeline corrosion monitoring. 

## 5. Further Discussions of the Limitations of the GA Prediction and Its Applicability for Practical Use

The proposed hybrid modeling strategy using CFD and GA is an efficient tool that is capable of dealing with complex problems. Importantly, the detection and monitoring of the internal corrosion of pipelines are often limited by field conditions, and thus, engineers usually choose a more conservative coverage for sensor deployment, to ensure the safety of the piping system. Based on this, the data-driven methods allow the extraction of the key features of complex problems and define the ultimate goal to achieve. Consequently, it can approximate field conditions with high accuracy by considering various factors and further facilitate the solving process of most engineering problems. To address its practical use for engineering applications, the limitations and applicability are discussed in the following:

(1) The core of the hybrid modeling strategy is to utilize the parametric analysis capability of CFD program, thus providing diverse data for GA prediction. For example, the topological changes in pipe fittings result in different flow patterns under different oil contents and flow velocities, which in turn lead to shear stress changes at critical locations along the piping system. This work selected three typical pipe fittings as prototypes to demonstrate the modeling applicability when solving engineering problems. The hybrid modeling workflow can be applied to different segments of the pipeline network, which is of interest to engineers, and the final layout of the sensor placement can be optimized with following the proposed strategy;
(2) On the other hand, in practical engineering, it is necessary to take the computational cost into account. The meshed elements in the CFD modeling are encoded in a single chromosome. It will lead to a large computational resource requirement when the number of elements is huge. Toward this point, researchers have addressed efforts on formatting the chromosome in pre-treatment of the algorithm to control the chromosome length [44,45]. For example, Ni et al. [46] used variable chromosome length optimization as an improved GA model to promote the adaptability of robot path finding. The chromosome format and length optimization are efficient ways to reduce the time complexity of the GA evolutions. Additionally, the probabilities of crossover and mutation are pre-determined as suggested in previous work. It was reported that the ratio of the probability of mutation over the probability of crossover had significant influence on the calculation efficiency [34] and the ultimate accuracy of optimization [47]. Further parametric study is necessary to investigate such impacts on the GA prediction;
(3) The most critical index for the GA prediction refers to the fitness function. The definition of the fitness in this work involves three primary factors, the time domain (time step *i*) of the fluid flow passing through the pipeline, critical locations (element position identification number *j*) that is likely to be subject to high corrosion rates, as well as the cost of sensors (parameter *b* associated with *j*). It is worth noting that if the sensor layout scheme contains different types of sensors, the advantage of the definition of the fitness function is that the overall cost can be fully considered, and the final scheme will be very beneficial to engineering decision-making. Therefore, the GA prediction in this work can also be applied when it is necessary to compare the economic benefits and sensing efficiencies of different types of corrosion sensors.

## 6. Conclusions and Future Work

This study introduced a hybrid modeling strategy using CFD and GA for optimization of the internal corrosion sensor placement, to achieve a good balance between economical cost and measurement accuracy. The methodology mainly consists of CFD analysis, corrosion rate prediction, and GA optimization. The complete workflow of the hybrid modeling was presented and illustrated through case studies involving three typical pipe fittings. Specifically, the conclusions can be drawn in the following:

- The essence of the hybrid modeling is to utilize the strong fluid analysis ability of CFD to provide a rich database and the stochastic searching ability of GA to explore optimal solutions on a global level. The information binding of CFD and GA is realized by converting the grid elements and time steps into chromosome length and population size, respectively. Importantly, the fitness function involves the in-line corrosion induced by the oil-water phase flow and overall cost of sensors. Based on this, the population evolution keeps iterating until the target fitness is achieve. It is an efficient way to find out the optimal scheme of sensor placement, especially for decision-making in engineering projects;
- The GA scheme used the field of corrosion rates as the original population input. Three typical pipe fittings were investigated, including the U-shaped, upward-inclined, and downward-inclined pipes. By adopting the suggested meshing strategy discussed in Section 2.2.5, the appropriate number of elements for the three scenarios were determined to 13,192, 8841, and 8841, respectively. After the GA optimizations, the sensor layout schemes were suggested to be a total of 6, 9, and 8, respectively. The best fitness and average fitness values, surpassing 0.9, intimately align with the core objectives of sensor placement optimization. These fitness metrics directly quantify the success of the strategy in identifying optimal sensor locations. The best fitness value signifies exceptional performance, pinpointing prime positions for corrosion risk mitigation. The average fitness value reflects the overall quality of selected solutions. Their congruence with optimization objectives highlights the strategy’s efficacy in striking a vital equilibrium between measurement accuracy and economic feasibility. This achievement is pivotal, enabling industries to access precise corrosion data while minimizing costs linked to complex investigations and monitoring setups. The elevated fitness values affirm the strategy’s capability to comprehensively address corrosion risks, underscoring its practical significance in enhancing pipeline integrity management. Additionally, scenario studies of various oil fraction and fluid velocity reveal that the high fluid velocity significantly reduces the optimized sensor quantity, focusing on the critical locations with high corrosion rates.

In order to further utilize the robust mapping ability of artificial intelligence, cross-integration with other algorithms can be further investigated, such as the Artificial Neural Network (ANN) integrated with GA and Particle Swarm Optimization (PSO)-ANN. Such data-driven approaches have been reported to yield good convergence and high accuracy when applied in engineering projects. Cross-integration with algorithms like ANN and PSO augments the proposed modeling strategy’s robustness. Integrating ANN can harness its mapping ability to complement GA’s optimization, yielding more accurate sensor placements. This fusion could overcome challenges like local convergence and enhancing reliability. PSO’s global search capabilities can counter local optima, further refining the sensor layout. In scenarios requiring nonlinear relationships or intricate search spaces, ANN-PSO synergy could excel. These integrations address limitations, bolstering feasibility studies and providing comprehensive solutions for complex sensor placement challenges in pipeline corrosion monitoring. In addition, due to the fact that the literature lacks details in sensor placement and limitations in performing field testing as analyzed, the effectiveness of the developed sensor placement scheme was not compared with other methods in this study. In the future, experimental and field testing are needed to validate the developed method through comparisons with other methods available in the literature.

## Figures and Tables

**Table 1:** Hydrodynamic properties of oil and water phase at 40 °C.

Item	Conoco LVT200 Oil	CO_2_ Dissolved Water
Density (kg/m^3^)	914	1000
Viscosity (Pa·S)	0.002	0.001
Interfacial tension (N/m)	0.032	0.072
Vapor pressure (kPa)	64.3	7.3
API weight	23	\

**Table 2:** Maximum shear stresses under different meshes.

No.	Global Element Size (m)	Elbow Element Size (m)	Number of Elements	Maximum Wall Shear Stress (Pa)
1	0.015	0.010	211,580	4.2670
2	0.020	0.015	90,288	4.2455
3	0.023	0.018	53,108	3.9888
4	0.025	0.020	45,220	3.9099
5	0.030	0.025	28,116	3.6448

**Table 3:** Critical parameters of the GA for scenario study.

Item	Symbol	Case 1	Case 2 and Case 3
Number of generations	*G*	1000	1000
Population size	*S*	1000	1000
Probability of crossover	*p_c_*	0.7	0.7
Probability of mutation	*p_m_*	0.1	0.1
Length of chromosome	*n*	13,192	8841
Time complexity (×10^6^)	*T*	22.8	1.53

**Table 4:** Measured corrosion rates derived from [30].

Flow Velocity (m/s)	Oil Content (%)	Measured Corrosion Rate (mm/yr)
0.28	0	3.00
0.28	20	4.00
0.28	60	4.30
0.28	80	1.00
1	0	4.25
1	20	5.40
1	60	5.75

**Table 5:** Location coordinates of potential sensor spots with CR specified.

Point No.	Case 1		Case 2		Case 3	
	(x, y, z)	CR (mm/yr)	(x, y, z)	CR (mm/yr)	(x, y, z)	CR (mm/yr)
1	(3.1468, −0.1396, −0.0093)	6.5805	(0.1432, −0.0091, 0.0972)	6.1531	(0.1262, −0.0435, 0.0874)	6.1694
2	(3.1396, −0.1468, −0.0093)	6.5799	(0.1262, −0.0091, 0.0972)	6.1528	(0.1093, −0.0435, 0.0874)	6.1689
3	(3.1545, −0.1328, −0.0093)	6.5794	(0.1431, −0.0267, 0.0939)	6.1524	(0.1430, −0.0435, 0.0874)	6.1686
4	(3.1328, −0.1545, −0.0093)	6.5780	(0.1598, −0.009, 0.0972)	6.1523	(0.0925, −0.0435, 0.0874)	6.1669
5	(3.1627, −0.1266, −0.0093)	6.5759	(0.1262, −0.0267, 0.0939)	6.1521	(0.1598, −0.0435, 0.0874)	6.1668
6	(3.1266, −0.1627, −0.0093)	6.5755	(0.1598, −0.0267, 0.0939)	6.1516	(0.1262, −0.0588, 0.0779)	6.1657
7	(3.1209, −0.1712, −0.0093)	6.5722	(0.1093, −0.0091, 0.0972)	6.1513	(0.1262, −0.0267, 0.0939)	6.1656
8	(3.1712, −0.1209, −0.0093)	6.5691	(0.1766, −0.0091, 0.0972)	6.1507	(0.1430, −0.0588, 0.0779)	6.1652
9	(3.1157, −0.1801, −0.0093)	6.5684	(0.1093, −0.0267, 0.0939)	6.1506	(0.1430, −0.0267, 0.0939)	6.1651
10	(3.1464, −0.1387, 0.0181)	6.5664	(0.1766, −0.0267, 0.0939)	6.1501	(0.1093, −0.0588, 0.0779)	6.1650

**Table 6:** GA-optimized sensor array locations.

	Case 1: 6 Locations		Case 2: 9 Locations		Case 3: 8 Locations	
Sensor No.	(x, y, z)	CR (mm/y)	(x, y, z)	CR (mm/y)	(x, y, z)	CR (mm/y)
1	(2.1324, −0.8458, −0.0115)	6.565	(0.1093, 0.0939, −0.0267)	6.1506	(2.5771, −0.2248, −0.0267)	6.1577
2	(3.1445, −0.1371, −0.0272)	6.5445	(0.1430, 0.0972,0.0090)	6.15	(0.1262, 0.0972, −0.0090)	6.1567
3	(2.18, −0.8845, −0.0104)	6.5412	(0.1431, 0.0874,0.0435)	6.1384	(2.5739, −0.2304, −0.0435)	6.1556
4	(3.1473, −0.14, 0.0045)	6.5410	(2.6807, 0.2808, −0.009)	6.1283	(0.1093, 0.0972, −0.0090)	6.1554
5	(2.1613, −0.8754, 0.0233)	6.5387	(2.6516, 0.264, −0.009)	6.1282	(2.5496, −0.2051, −0.009)	6.1547
6	(3.1333, −0.155, 0.0045)	6.5378	(2.637, 0.2556, −0.009)	6.1281	(0.1262, 0.0514, −0.0830)	6.1537
7			(2.6953, 0.2892,0.009)	6.1280	(2.5156, −0.1968, −0.0435)	6.1528
8			(2.7390, 0.3145, −0.009)	6.1277	(2.6079, −0.2388, 0.0090)	6.1507
9			(2.6225, 0.2472, −0.009)	6.1276		

**Table 7:** Total cost of sensor placement plans for different methods (USD).

Maximum Allowable Corrosion Rate (mm/yr)	CFD-GA Method	CFD without GA Optimized
2.00	550	3,250
3.00	550	3,000
4.00	500	2,100
5.00	350	850

## Data Availability

Not applicable.
